# Long-term outcomes following the clipping of ruptured intracranial aneurysms of the anterior circulation: A retrospective institutional study

**DOI:** 10.3892/mi.2025.257

**Published:** 2025-07-31

**Authors:** Diamantoula Pagkou, George Fotakopoulos, Evangelos Kogias, Kiriaki Papadopoulou, Ioannis Patsalas, Nikolaos Foroglou

**Affiliations:** Department of Neurosurgery, AHEPA University Hospital, Aristotle University of Thessaloniki, 54636 Thessaloniki, Greece

**Keywords:** aneurysmal subarachnoid hemorrhage, microsurgical clipping, anterior circulation aneurysms, timing of surgery, clinical outcomes

## Abstract

Aneurysmal subarachnoid hemorrhage (SAH) constitutes a devastating and life-threatening neurosurgical emergency. Over a number of years, there has been a debate as regards the most suitable timing for surgery. The present retrospective study aimed to investigate the association between the timing of treatment and the outcomes of patients who underwent clipping of ruptured intracranial aneurysms. The present study performed a retrospective analysis of 92 of 142 consecutive patients who were diagnosed with anterior circulation ruptured aneurysms and treated only by microsurgical clipping between January, 2013 and December, 2018. The patients were divided into two groups, namely group A, which included patients who underwent microsurgical clipping for ruptured anterior circulation aneurysm in the early stages (within the first 3 days of aneurysm rupture occurrence), and group B, which included those who were operated on in the late stages (after 3 days of aneurysm rupture occurrence). The age of the patients ranged from 26 to 78 years, with a mean age of 54.6 years. In total, 52 patients were female (56.6%) and 40 patients were male (43.4%). Risk factors associated with a high incidence of rupture included hypertension in 47 (51.0%) patients, cigarette smoking in 39 (42.3%) patients, alcohol consumption in 20 (21.7%) patients and diabetes mellitus in 2 (2.1%) patients. The present study revealed that the mortality rate was lower in group B (P=0.011; 4.3% compared with 11.9% in group A). This indicates that particularly the timing of surgery in group B (patients who were operated on in the late stages, after 3 days of aneurysm rupture occurrence) was more appropriate. In addition, no complications, post-operative hemiplegia and mortality were all independent factors associated with good outcomes (≥2 modified Rankin scale) (P<0.05 for all three parameters). On the whole, the present study demonstrates that the outcomes of patients who underwent microsurgical clipping for ruptured anterior circulation aneurysms ≥3 days after admission or rupture occurrence, exhibited favorable outcomes compared to patients operated on during the first 2 days of occurrence.

## Introduction

Aneurysmal subarachnoid hemorrhage (SAH) constitutes a devastating and life-threatening neurosurgical emergency with an estimated global incidence of 9.1 per 100,000 individuals per year (1,2). Due to its catastrophic short-term complications, SAH remains a major cause of morbidity and mortality worldwide, even though novel diagnostic and treatment techniques have been developed over the past decades. Indeed, during the early stages of aneurysmal SAH, there is a high risk of re-bleeding, hydrocephalus, intracerebral hematoma, generalized cerebral edema and vasospasm, while long-term complications include delayed cerebral ischemia (DCI) (3-5).

The severity of the clinical presentation upon admission, re-bleeding, the timing of the surgery and the type of treatment are predictors of neurological outcomes and SAH-related disability (6,7). Furthermore, patients with higher Hunt and Hess grades (grades 4 and 5) upon admission, particularly the elderly, are more likely to have poor clinical outcomes (8).

Upon the diagnosis of a ruptured intracranial aneurysm, the main goal is to secure the aneurysm as soon as possible to prevent re-bleeding. Before the 1990s, microsurgical clipping was the only available treatment option for ruptured intracranial aneurysms and while it still confers satisfactory long-term results regarding re-operation rates, it is characterized by high peri-operative complications and long periods of hospitalization (4,5,9,10). On the other hand, the introduction of endovascular treatment has led to lower peri-interventional complication rates; however, this is associated with a higher rate of incomplete occlusion and in-hospital case fatalities (11,12).

The timing of the surgery plays a crucial role concerning the risk of re-bleeding and mortality rates, particularly in patients with a good clinical condition upon admission (13-16). Over a number of years, there has been a debate as regards the most suitable timing of surgery (17,18). The early clipping of the cerebral aneurysm may permit for the secure preface of hypertensive management prevented at the development and/or treatment of DCI (14). On the other hand, delayed surgical management may decrease the morbidity and mortality of the microsurgical intervention due to improvements in surgical conditions and the general condition of individual patients, mainly in those with higher Hunt and Hess grades (grades 4 and 5) upon admission (13).

The present retrospective study aimed to investigate the association between the timing of treatment and the outcomes of patients who underwent the clipping of ruptured intracranial aneurysms. The present study and also aimed to provide contemporary data for a more balanced approach when assessing the optimal treatment strategy for a given aneurysm, microsurgical vs. endovascular, when both options are available.

## Materials and methods

Patient population and aneurysm characteristics. A retrospective analysis of 92 of 142 consecutive patients who were diagnosed with anterior circulation ruptured aneurysms and treated only by microsurgical clipping was conducted between January, 2013 and December, 2018 (AHEPA University Hospital, Aristotle University School of Medicine, Thessaloniki, Greece).

The study protocol was approved by the Ethics Committee of the hospital-(AHEPA University Hospital, Aristotle School of Medicine Thessaloniki, Greece; Ref. no. 1085-2023). In compliance with the current legislation, the National Data Protection Authority was notified on its conduction (Ref. no. 985-2024). The study was conducted in accordance with the ethical standards laid down in the Declaration of Helsinki 1964 and its later amendments. Written informed consent was obtained from all included patients or their next-of-kin prior to surgery. All patients were >18 years of age.

**Figure 1:**
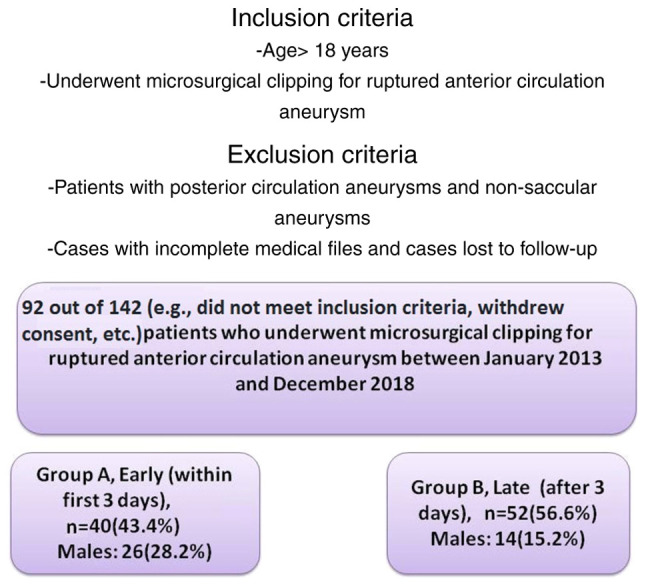
Flow chart of the inclusion process for the participants in the present study.

Patients with posterior circulation aneurysms and non-saccular aneurysms were excluded from the study. In the final pool, 92 patients were included, and these patients were divided into two groups. Data collection was performed, and the data were reviewed and analyzed by two physicians (GF and DP) based on the following inclusion criteria: Patients aged >18 years who underwent microsurgical clipping for ruptured anterior circulation aneurysm between 2013 and 2018. Cases with incomplete medical files and cases lost to follow-up were excluded (Fig. 1).

Demographic data including age, sex, comorbidities and post-operative outcomes were collected prospectively. Baseline characteristics included age, sex, risk factors, aneurysm size and location, clinical presentation upon admission, Fisher grade, and the timing of surgery (Table I). Risk factors for aneurysm formation and rupture, including hypertension, diabetes mellitus, hypercholesterolemia and smoking were documented and the data are also presented in Table I. The clinical presentation of the patient upon admission was assessed according to Hunt and Hess grading scale (Table I). Clinical status upon admission was dichotomized as good (grade ≤2) and severe (grade ≥3). At the emergency department, all patients underwent urgent Computed Tomography (CT) angiography. Aneurysm location, size, and extent of SAH according to Fisher grading scale were documented and the presence of multiple aneurysms was also investigated (Table I). The timing of the surgery was dichotomized as early (within the first 3 days after SAH) and late (after ≥4 days).

### Clinical data

The patients were divided into two groups, namely group A, which included patients who underwent microsurgical clipping for ruptured anterior circulation aneurysm in the early stages (within the first 3 days of aneurysm rupture occurrence), and group B, which included those who were operated on in the late stages (after 3 days of aneurysm rupture occurrence).

### Perioperative treatment and surgical technique

During hospitalization, all patients received the same medical treatment protocol [nimodipine, 60 mg (as 2x30 mg capsules) every 4 h for 21 consecutive days, antihypertensives to control blood pressure, pain relievers (acetaminophen, 500 mg every 8 h for headache) and antiseizure medications (levetiracefan, 500 mg every 12 h]. Standard pterional craniotomy was used for the clipping of anterior circulation aneurysms. In some cases, temporary clip application was performed. A small number of patients underwent decompressive craniectomy due to diffuse cerebral edema and massive intracerebral hemorrhage and some of them underwent ventriculoperitoneal shunt placement for hydrocephalus. Following surgery, patients were moved to the intensive care unit.

### Follow-up and clinical outcomes

The presence of pre-operative re-bleeding, hydrocephalus and diffuse cerebral edema was assessed. The modified Rankin scale (mRS) was used to assess clinical outcomes. According to the mRS, patients were categorized as mRS ≥2 (good outcome) and mRS <2 (poor outcome). The mRS is a severity-of-disease classification system that estimates the level of morbidity during the everyday actions of participants who have suffered a stroke or have other diseases leading to neurological disability. A numerical score from 0 to 6 was assigned based on several factors, with higher scores indicating a more severe situation and a greater risk of mortality (19). Post-operative outcomes were evaluated no less than 12 months following surgical management.

### Statistical analysis

Statistical analyses were carried out using the Statistical Package for the Social Sciences (SPSS 11; SPSS, Inc.). The normality of the distribution of variables was evaluated using the Shapiro-Wilk test. Categorical variables were compared between groups using Fisher's exact test. The continuous records also were assessed using the Mann-Whitney U test. Receiver operating characteristic (ROC) analysis was applied to expose the causes that are related to patients who were diagnosed with anterior circulation ruptured aneurysms and treated only by microsurgical clipping and affect the outcomes of those patients. A P-value <0.05 was considered to indicate a statistically significant difference.

## Results

### Patient and aneurysm characteristics

The baseline characteristics of patients and aneurysms are outlined in Table I. The present study included 92 patients, admitted to the emergency department with a high clinical suspicion of SAH, which was confirmed radiologically, between January, 2013 and December, 2018. A total of 92 anterior circulation ruptured aneurysms were identified following computed tomography angiography. All patients underwent microsurgical clipping of the aneurysm.

The age of the patients ranged from 26 to 78 years, with a mean age of 54.6 years. In total, 52 patients were female (56.6%) and 40 patients were male (43.4%). Risk factors associated with a high incidence of rupture included hypertension in 47 (51.0%) patients, cigarette smoking in 39 (42.3%) patients, alcohol consumption in 20 (21.7%) patients and diabetes mellitus in 2 (2.1%) patients (Table I).

The most common rupture site was the anterior communicating artery in 49 (53.2%) cases followed by ruptured middle cerebral artery aneurysms in 22 (23.9%) cases. In total, 11 cases (11.9%) had more than one aneurysm. As regards the aneurysm diameter, the majority of them were small [0-4.9 mm; 27 patients (29.3%)] and medium [5-14.9 mm; 60 patients (65.2%)] in size (Table I).

Upon admission, the majority of cases (60 patients, 65%) presented with favorable Hunt and Hess grading scale scores (grades 1-3), while the remaining 32 (35%) patients were categorized as grades 4 and 5. A total of 29 (31.5%) patients had Fisher grade 2 at the initial presentation, 33 (35.8%) patients had Fisher grade 3, and the remaining 30 patients had grade 4 (32.6%) (Table I). In terms of the timing of surgery, 40 patients underwent microsurgical clipping during the first 3 days of hospitalization (43.4%) and the remaining 52 (56.6%) patients were operated on after 3 days (Table I).

### Association between factors and clinical outcomes

Univariate analysis revealed that there was a statistically significant difference in the no complications, post-operative hemiplegia, diffuse edema/decompressive craniectomy, re-bleeding, Fisher grading scale, Hunt and Hess grading scale and mortality between the participants who underwent microsurgical clipping during the first 3 days of hospitalization (group A) and those that underwent surgery after 3 days (group B) (P<0.05, Table II).

Multivariate analysis (Table III) revealed that no complications, post-operative hemiplegia and mortality were all independent factors associated with good outcomes (≥2 mRS) (P<0.05 for all three parameters).

**Figure 2:**
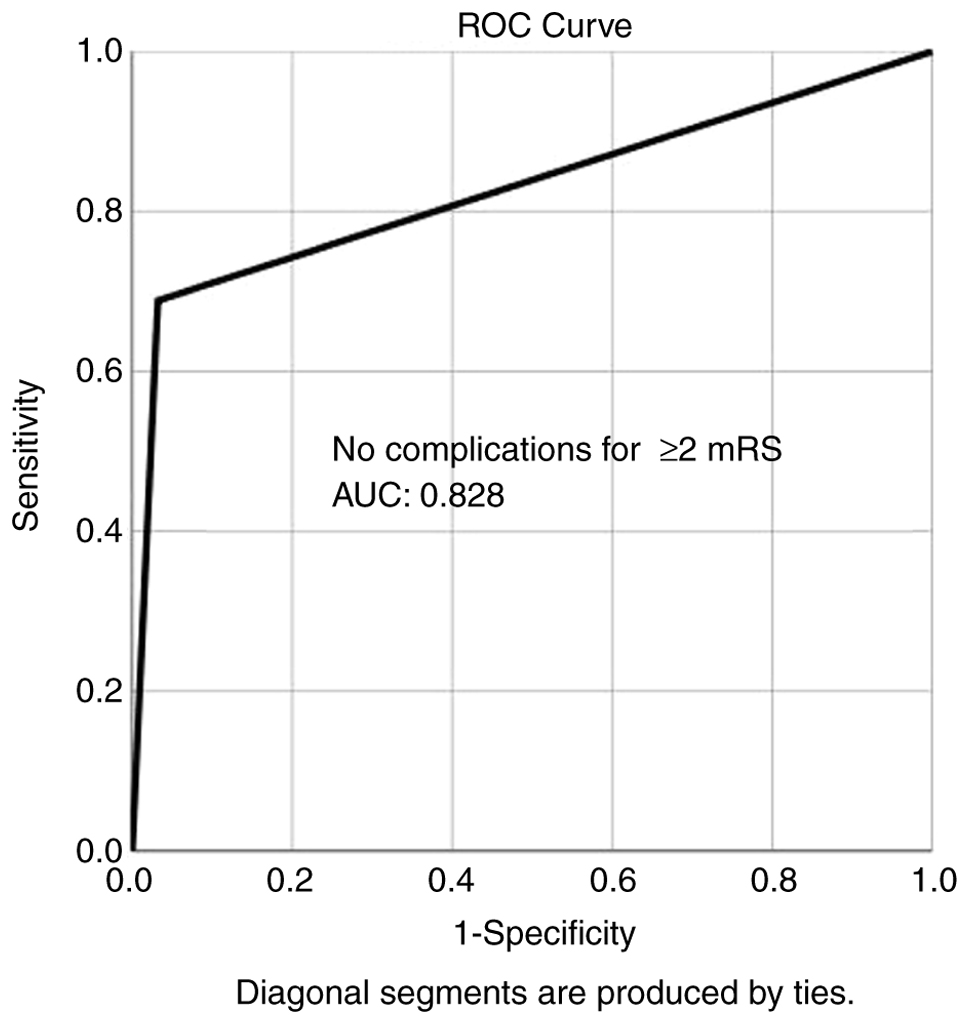
ROC curve for no complications, predicting good outcome (>2 mRS) during follow-up. AUC, 0.828. ROC, receiver operative characteristic; AUC, area under the curve.

**Figure 3:**
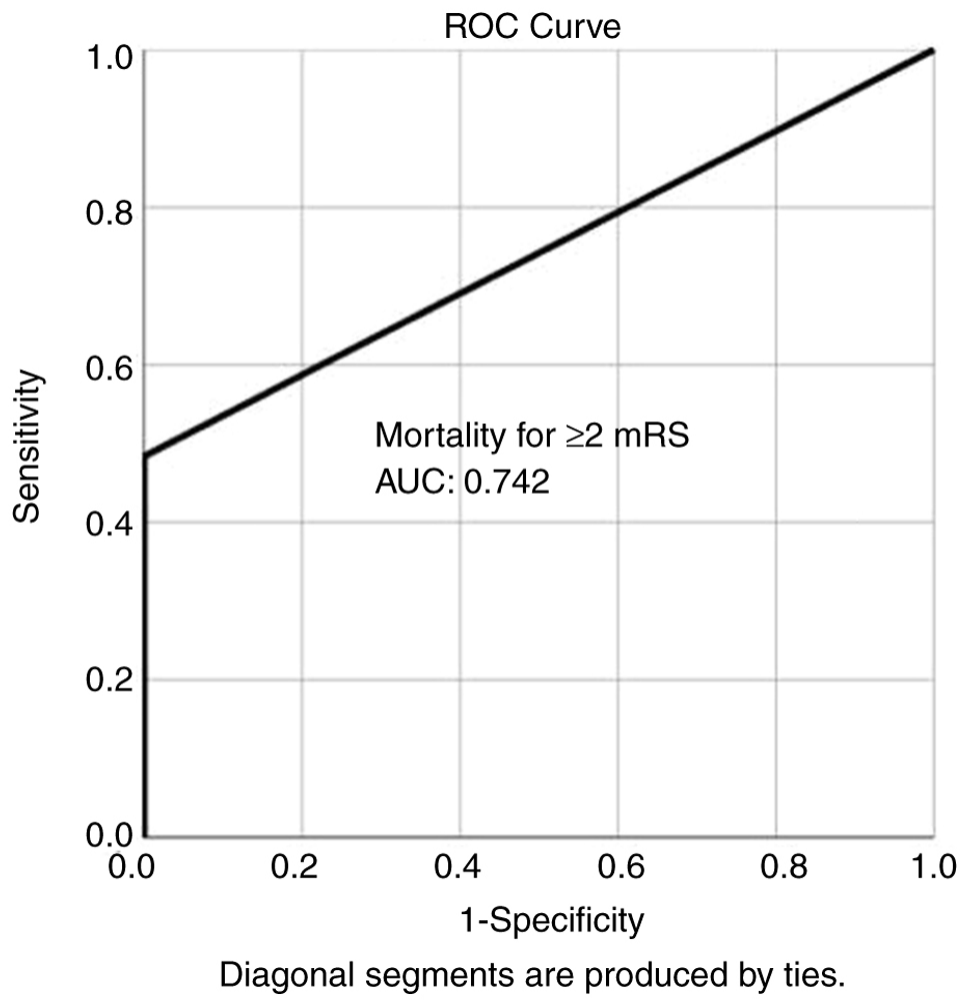
ROC curve for mortality, predicting good outcome (≥2 mRS) during follow-up. AUC, 0742. ROC, receiver operative characteristic; AUC, area under the curve.

Overall, ROC analysis demonstrated that no complications exhibited the optimal performance to predict good outcome (≥2 mRS), as evaluated by an area under the curve standard error [AUC (SE)] of [0.828 (0.043) and (P=0.000)] (Table III and Fig. 2). In addition, ROC analysis demonstrated that, among the variables, mortality was lower in group B compared with group A, indicating the superiority of group B compared with group A, as evaluated by an AUC (SE) of 0.742 (0.062) and P=0.000 (Table III and Fig. 3).

## Discussion

The results of the present study demonstrated that the outcomes of patients who underwent microsurgical clipping for ruptured anterior circulation aneurysms ≥3 days after admission or rupture occurrence, exhibited favorable outcomes compared to patients operated on during the first 2 days of occurrence. The good clinical condition concerning SAH-related complications (no complications); the development of post-operative hemiplegia related to DCI, and mortality were all independent factors associated with good outcomes (≥2 mRS). In addition, even though the majority of the cases (78.4%) were admitted with Fisher grading scale 3 or 4, in the present study the mortality rate was 16.3%. On the other hand, risk factors for aneurysm formation and rupture, including hypertension, diabetes mellitus, hypercholesterolemia and smoking were not statistically significant parameters reinforcing the initial hypothesis that the timing of the surgery plays a role in patient outcomes.

Early surgery, within the first 3 days, seems to be associated with lower likelihood of a positive outcome, especially in those patients in good clinical condition on admission [13]. For this reason, most centers now intend to operate on an aneurysm within two days of the SAH (20,21). However, microsurgical management for aneurysmal SAH may be more dangerous during this two-day interval, and thus treatment management must comprise contemplations concerning the risk of re-hemorrhage (20). In addition, for these patients, who are only suitable for surgery three days after SAH, it is significant to identify the most favorable timing for aneurysm clipping occlusion, i.e. whether it should be until 14 days after SAH (22). In the present study, surgical morbidity and mortality, between patients submitted to early, intermediate, or late surgery, was statistically significant, with patients who underwent surgical aneurysm repair in an interval ≥3 days indicating a more favorable outcome.

On the other hand, there were studies that the timing of surgery did not have a significant role in surgical outcomes, apart from clinical condition on admission (15,23). Additional subgroup analysis proposed a tendency that recommended greater advantages with early intervention, which, in other words, encouraged a policy of surgery as early as possible (23). In addition, based on these results, it was recommended that clipping between five and ten days after the event of aneurysmal rupture did not lead to a higher possibility of DCI (22). Instead, operating on the swollen and vulnerable brain tissue led to high rates of perioperative complications (24). The present study demonstrated that clinical outcomes were improved when aneurysm occlusion was performed at a later stage after SAH after differences. It is imperative to understand that patients were not randomized for the timing of the surgery, and the unfavorable outcome in the group of patients operated on in the early stages, was possibly associated with the causes of the post-operative condition, such as a poor clinical condition on admission, early re-hemorrhage, or early worsening from other reasons. Notably, in the present study, re-bleeding did not occur more often in patients operated on after day 3, and this is a critical factor when deciding on patients' management.

A previous study on endovascular management report that the embolization of ruptured intracranial aneurysms can be applied safely to patients who were admitted between post-hemorrhage days 4 and 10, with no significant increase in mortality, and regardless of a higher risk of vasospasm at the time of treatment (25). In the present study, the mortality rate was lower in those patients who were operated on 3 days after aneurysm rupture occurrence.

The present study had several limitations, such as that it was a retrospective study of medical records analysis and was performed in a single center with a limited number of cases. In addition, the neurological outcomes of patients with SAH is dependent on the underlying initial pathology and the subsequent vasospasm. Another limitation was the restrictions concerning the lack of inclusion of patients presenting with a worse overall clinical condition.

In conclusion, the present study revealed that the outcomes of patients who underwent microsurgical clipping for ruptured anterior circulation aneurysms ≥3 days after admission or rupture occurrence, exhibited favorable outcomes compared to patients operated on during the first 2 days of occurrence. The good clinical condition concerning SAH-related complications (no complications), the development of post-operative hemiplegia related to DCI and mortality were all independent factors associated with good outcomes. In addition, even though the majority of the cases were admitted with Fisher grading scale scores of 3 or 4, in the present study, the mortality rate was 16.3%. Although the endovascular treatment of anterior circulation ruptured aneurysms, during the past years, has been the treatment of choice following SAH, clipping may be considered a valid alternative.

## Figures and Tables

**Table I:** Baseline demographic characteristics of the patients.

Parameters	All patients, n=92 (100%)	Group A, early (within first 3 days), n=40 (43.4%)	Group B, late (after 3 days), n=52 (56.6%)	P-value
Age, mean ± SD (years)	54.6±11.5	52.2±13.2	56.5±9.7	0.197
Sex, n (%)				0.150
Male	40 (43.4)	26(65)	14(35)	
Female	52 (56.6)	14 (26.9)	38 (73.1)	
Alcohol consumption, n (%)				0.060
Yes	20 (21.7)	5 (5.4)	15 (16.3)	
No	72 (78.3)	35 (38.8)	37 (40.5)	
Diabetes, n (%)				0.851
Yes	2 (2.1)	1 (1.0)	1 (1.0)	
No	90 (97.9)	39 (42.5)	51 (55.4)	
Hypertension, n (%)				0.855
Yes	47(51)	20 (21.7)	27 (29.3)	
No	45(49)	20 (21.7)	25 (27.3)	
Smoking status, n (%)				0.195
Yes	39 (42.3)	20 (21.7)	19 (20.6)	
No	53 (57.7)	20 (21.7)	33(36)	
Aneurysm size, n (%)				0.015
Small (<5 mm)	27 (29.3)	17 (18.4)	10 (10.8)	
Medium (5-15 mm)	60 (65.2)	19 (20.6)	41 (44.5)	
Large (>15-25 mm)	4 (4.3)	3 (3.2)	1 (1.0)	
Giant (>25 mm)	1 (1.0)	1 (1.0)	0 (0)	
Complications^a^, n (%)				
No complication	43 (46.7)	21 (22.8)	22 (23.9)	0.331
Hydrocephalus	27 (29.3)	9 (9.7)	18 (19.5)	0.206
Post-operative hemiplegia	7 (7.6)	3 (3.2)	4 (4.3)	0.972
Pneumonia	6 (6.5)	0 (0)	6 (6.5)	0.026
Diffuse edema/DC	15 (16.3)	10 (10.8)	5 (5.4)	0.048
Multiorgan failure	4 (4.3)	2 (2.1)	2 (2.1)	0.788
Re-bleeding	10 (10.8)	5 (5.4)	5 (5.4)	0.659
Location^a^, n (%)				
Acom	49 (53.2)	20 (21.7)	29 (31.5)	0.582
ICA/Pcom	17 (18.4)	5 (5.4)	12 (13.0)	0.195
MCA	22 (23.9)	13 (14.1)	9 (9.7)	0.090
ICA-anterior choroidal	4 (4.3)	1 (1.0)	3 (3.2)	0.446
Multiple aneurysm	11 (11.9)	6 (6.5)	5 (5.4)	0.446
Fisher grading scale, n (%)				0.672
1	0 (0)	0 (0)	0 (0)	
2	29 (31.5)	12 (13.0)	17 (18.4)	
3	33 (35.8)	13 (14.1)	20 (21.7)	
4	30 (32.6)	15 (16.3)	15 (16.3)	
Hunt and Hess grading scale, n (%)				0.002
0	0 (0)	0 (0)	0 (0)	
1	31 (33.6)	20 (21.7)	11 (11.9)	
2	23(25)	4 (4.3)	19 (20.6)	
3	6 (6.5)	0 (0)	6 (6.5)	
4	26 (28.2)	13 (14.1)	13 (14.1)	
5	6 (6.5)	3 (3.2)	3 (3.2)	
Modified Rankin rading scale, n (%)				
Good outcome ≥2	61 (66.3)	24 (26.0)	37 (40.7)	
Poor outcome <2	31 (33.6)	16 (17.3)	15 (16.3)	-
Mortality, n (%)				0.011
Yes	15 (16.2)	11 (11.9)	4 (4.3)	
No	77 (83.8)	29 (31.5)	48 (52.3)	

^a^For the parameters Complications and ‘Location’, each subcategory was compared vs. the rest of the patients of that category. SD, standard deviation; DC, decompressive craniectomy; Acom, anterior communicating artery; ICA, internal carotid artery (ICA); Pcom, posterior communicating artery.

**Table II:** Univariate analysis for good outcomes (≥2 mRS).

Parameters	Good outcomes (≥2 mRS), n=61 (66.3%)	Poor outcomes (<2 mRS), n=31 (33.7%)	P-value
Groups, timing of surgery	8.2±6.0	6.5±5.8	0.202
Age, mean ± SD (years)	54.4±12.0	55.1±10.5	0.934
Sex, n (%)			0.117
Male	23 (57.5)	17 (42.5)	
Female	38(73)	14(27)	
Alcohol consumption, n (%)			0.500
Yes	12 (13.0)	8 (8.6)	
No	49 (53.2)	23(25)	
Diabetes, n (%)			0.622
Yes	1 (1.0)	1 (1.0)	
No	60 (65.2)	30 (32.6)	
Hypertension, n (%)			0.943
Yes	31 (33.6)	16 (17.3)	
No	30 (32.6)	15 (16.3)	
Smoking status, n (%)			0.407
Yes	24 (26.0)	15 (16.3)	
No	37 (40.2)	16 (17.3)	
Aneurysm size, n (%)			0.234
Small (<5 mm)	21 (22.8)	6 (6.5)	
Medium (5-15 mm)	38 (41.3)	22 (23.9)	
Large (>15-25 mm)	2 (2.1)	2 (2.1)	
Giant (>25 mm)	0 (0)	1 (1.0)	
Complications^a^, n (%)			
No complication	42 (45.6)	1 (1.0)	0.001
Hydrocephalus	14 (15.2)	13 (14.1)	0.059
Post-operative hemiplegia	2 (2.1)	5 (5.4)	0.028
Pneumonia	2 (2.1)	4 (4.3)	0.077
Diffuse edema/DC	3 (3.2)	12 (13.0)	0.001
Multiorgan failure	1 (1.0)	3 (3.2)	0.074
Re-bleeding	3 (3.2)	7 (7.6)	0.010
Location^a^, n (%)	-	-	-
Acom	34 (36.9)	15 (16.3)	0.504
ICA/Pcom	12 (13.0)	5 (5.4)	0.679
MCA	12 (13.0)	10 (10.8)	0.181
ICA-anterior choroidal	4 (4.3)	0 (0)	0.145
Multiple aneurysm	4 (4.3)	7 (7.6)	0.127
Fisher grading scale, n (%)			0.001
1	0 (0)	0 (0)	
2	29 (31.5)	0 (0)	
3	23(25)	10 (10.8)	
4	9 (9.7)	21 (22.8)	
Hunt and Hess grading scale, n (%)			0.001
0	0 (0)	0 (0)	
1	30 (32.6)	1 (1.0)	
2	17 (18.4)	6 (6.5)	
3	3 (3.2)	3 (3.2)	
4	9 (9.7)	17 (18.4)	
5	2 (2.1)	4 (4.3)	
Mortality, n (%)			0.001
Yes	0 (0)	15 (16.3)	
No	61	16 (17.3)	

^a^For the parameters Complications and ‘Location’, each subcategory was compared vs. the rest of the patients of that category. SD, standard deviation; DC, decompressive craniectomy; Acom, anterior communicating artery; ICA, internal carotid artery (ICA); Pcom, posterior communicating artery.

**Table III:** Multivariate analysis and ROC analysis of good outcomes (≥2 mRS).

A, Multivariate analysis				
			95% CI for Exp(B)	
Parameter	P-value	Exp(B)	Lower	Upper
No complication	0.005	0.300	0.086	0.482
Post-operative hemiplegia	0.007	-0.210	-0.647	-0.104
Diffuse edema/DC	0.183	-0.126	-0.399	0.077
Re-bleeding	0.757	0.025	-0.209	0.286
Fisher grading scale	0.099	-0.224	-0.289	0.025
Hunt and Hess grading scale	0.356	0.137	-0.054	0.149
Mortality	0.000	-0.422	-0.766	-0.321
B, ROC analysis				
Parameter	P-value	Area	Std. error	CI (95%) lower-upper
No complication	0.000	0.828	0.043	0.744-0.912
Post-operative hemiplegia	0.316	0.564	0.066	0.436-0.693
Mortality	0.000	0.742	0.062	0.621-0.863

SD, standard deviation; CI, conﬁdence interval; DC, decompressive craniectomy; ROC, receiver operative characteristic.

## Data Availability

The data generated in the present study may be requested from the corresponding author.

## References

[1] Steiner T, Juvela S, Unterberg A, Jung C, Forsting M, Rinkel G (2013). European stroke organization guidelines for the management of intracranial aneurysms and subarachnoid haemorrhage. Cerebrovasc Dis.

[2] D'Souza S (2015). Aneurysmal subarachnoid hemorrhage. J Neurosurg Anesthesiol.

[3] Petridis AK, Kamp MA, Cornelius JF, Beez T, Beseoglu K, Turowski B, Steiger HJ (2017). Aneurysmal subarachnoid hemorrhage. Dtsch Arztebl Int.

[4] Fotakopoulos G, Tsianaka E, Fountas K, Makris D, Spyrou M, Hernesniemi J (2017). Clipping versus coiling, in anterior circulation ruptured intracranial aneurysms: A meta-analysis. World Neurosurg.

[5] Fotakopoulos G, Andrade-Barazarte H, Tjahjadi M, Goehre F, Hernesniemi J (2021). Clipping versus coiling in ruptured basilar apex aneurysms: A meta-analysis. Turk Neurosurg.

[6] Tsolaki V, Aravantinou-Fatorou A, Georgakopoulou VE, Spandidos DA, Papalexis P, Mathioudakis N, Tarantinos K, Trakas N, Sklapani P, Fotakopoulos G (2022). Early diagnosis of cerebral vasospasm associated with cerebral ischemia following subarachnoid hemorrhage: Evaluation of computed tomography perfusion and transcranial doppler as accurate methods. Med Int (Lond).

[7] Fotakopoulos G, Makris D, Kotlia P, Kapsalaki E, Papanikolaou J, Georgiadis I, Zakynthinos E, Fountas K (2018). The value of computed tomography perfusion & transcranial Doppler in early diagnosis of cerebral vasospasm in aneurysmal & traumatic subarachnoid hemorrhage. Future Sci OA.

[8] Zhang Y, Zhu X, Hou K, Zhao J, Gao X, Sun Y, Wang W, Zhang X (2016). Clinical outcomes of surgical clipping for intracranial aneurysms in patients with a Hunt and Hess grade 4 or 5. Arq Neuropsiquiatr.

[9] Fotakopoulos G, Andrade-Barazarte H, Alexandros B, Hernesniemi J (2023). A meta-analysis of lateral supraorbital vs. mini pterional approach in the outcome of rupture and unruptured noncomplex aneurysms' surgery. Neurocirugia (Engl Ed).

[10] Fotakopoulos G, Georgakopoulou VE, Gatos C, Christodoulidis G, Foroglou N (2025). Microsurgery treatment as an optimal management of posterior cerebral artery aneurysms: A systematic review and meta-analysis. Cureus.

[11] Mohammad F, Horiguchi T, Mizutani K, Yoshida K (2020). Clipping versus coiling in ruptured aneurysms of the anterior cerebral circulation. Open J Mod Neurosurg.

[12] Ahmed SI, Javed G, Bareeqa SB, Samar SS, Shah A, Giani A, Aziz Z, Tasleem A, Humayun SH (2019). Endovascular coiling versus neurosurgical clipping for aneurysmal subarachnoid hemorrhage: A systematic review and meta-analysis. Cureus.

[13] Dellaretti M, Batista DM, de Almeida JC, de Souza RF, Ronconi DE, de Almeida CER, Fontoura RR, Júnior WF (2018). Surgical treatment of ruptured intracranial aneurysms: Timing of treatment and outcome. Interdiscip Neurosurg.

[14] Yao Z, Hu X, Ma L, You C, He M (2017). Timing of surgery for aneurysmal subarachnoid hemorrhage: A systematic review and meta-analysis. Int J Surg.

[15] Weil AG, Zhao JZ (2012). Treatment of ruptured aneurysms: Earlier is better. World Neurosurg.

[16] Duangthongphon P, Souwong B, Munkong W, Kitkhuandee A (2019). Results of a preventive rebleeding protocol in patients with ruptured cerebral aneurysm: A retrospective cohort study. Asian J Neurosurg.

[17] Drake CG (1981). Progress in cerebrovascular disease. Management of cerebral aneurysm. Stroke.

[18] Nieuwkamp DJ, de Gans K, Algra A, Albrecht KW, Boomstra S, Brouwers PJAM, Groen RJM, Metzemaekers JDM, Nijssen PCG, Roos YBWEM (2005). Timing of aneurysm surgery in subarachnoid haemorrhage-an observational study in The Netherlands. Acta Neurochir (Wien).

[19] Broderick JP, Adeoye O, Elm J (2017). Evolution of the modified rankin scale and its use in future stroke trials. Stroke.

[20] Mahaney KB, Todd MM, Torner JC (2011). Variation of patient characteristics, management, and outcome with timing of surgery for aneurysmal subarachnoid hemorrhage. J Neurosurg.

[21] Chyatte D, Fode NC, Sundt TM Jr (1988). Early versus late intracranial aneurysm surgery in subarachnoid hemorrhage. J Neurosurg.

[22] Dorhout Mees SM, Molyneux AJ, Kerr RS, Algra A, Rinkel GJE (2012). Timing of aneurysm treatment after subarachnoid hemorrhage: Relationship with delayed cerebral ischemia and poor outcome. Stroke.

[23] Zhao C, Wei Y (2017). Surgical timing for aneurysmal subarachnoid hemorrhage: A meta-analysis and systematic review. Turk Neurosurg.

[24] Kassell NF, Drake CG (1982). Timing of aneurysm surgery. Neurosurgery.

[25] Lawson MF, Chi YY, Velat GJ, Mocco JD, Hoh BL (2010). Timing of aneurysm surgery: The international cooperative study revisited in the era of endovascular coiling. J Neurointerv Surg.

